# Application of Population Balance Models in Particle-Stabilized Dispersions

**DOI:** 10.3390/nano13040698

**Published:** 2023-02-11

**Authors:** Susanne Röhl, Lena Hohl, Sebastian Stock, Manlin Zhan, Tobias Kopf, Regine von Klitzing, Matthias Kraume

**Affiliations:** 1 Department of Chemical and Process Engineering, Technische Universität Berlin, 10623 Berlin, Germany; 2 Institute for Condensed Matter Physics, Technische Universität Darmstadt, 64289 Darmstadt, Germany

**Keywords:** Pickering emulsion, stirred tank, interface coverage degree, coalescence efficiency

## Abstract

In this study, a first approach to model drop size distributions in agitated nanoparticle-stabilized liquid/liquid systems with population balance equations is presented. Established coalescence efficiency models fail to predict the effect of steric hindrance of nanoparticles at the liquid/liquid interface during the film drainage process. A novel modified coalescence efficiency is developed for the population balance framework based on the film drainage model. The elaborate submodel considers the desorption energy required to detach a particle from the interface, representing an energy barrier against coalescence. With an additional implemented function in the population balance framework, the interface coverage rate by particles is calculated for each time step. The transient change of the coverage degree of the phase interface by particles is thereby considered in the submodel. Validation of the modified submodel was performed with experimental data of agitated water-in-oil (w/o) dispersions, stabilized by well-defined spherical silica nanoparticles. The nanospheres with a size of 28 nm are positively charged and were hydrophobized by silanization with dimethyloctadecyl[3-(trimethoxysilyl)propyl]ammoniumchloride. This modeling approach is a first step toward predicting time-resolved dynamic drop size distributions of nanoparticle-stabilized liquid/liquid systems.

## 1. Introduction

Solid particles ranging in size from nanometers to micrometers can be used to stabilize dispersions against coalescence. The dispersions can be prepared via ultrasonication [1] or in agitated tanks [2]. Moreover, combining solid and soft particles to stabilize dispersions is a promising novel approach [3]. There are numerous research projects on applying particle-stabilized systems, for example, in the food industry [4] or in the field of innovative catalytic reactions [5] including separation technologies [6]. In this context, knowledge and prediction of the available liquid/liquid interface is crucial to design and optimize processes with particle-stabilized liquid/liquid dispersions.

This work aims to describe the coalescence inhibition induced by particles in agitated liquid/liquid systems and predict drop size distributions using population balance equations (PBEs).

Despite the higher numerical effort by solving the population balance equations compared to the use of established (semi)empirical Weber correlations [7], PBEs exhibit decisive advantages as a modeling approach. Complete and transient drop size distributions (DSDs) can be modeled with PBEs beside single characteristic diameters in steady state (e.g., Sauter mean diameters). An additional advantage is the modular concept of the PBE, which offers easy implementation and modification of submodels for the respective description of breakage and coalescence processes. The successful application of population balances to predict the drop size distribution of agitated liquid/liquid systems has been shown in the work by Coulaloglou and Tavlarides (C&T) [8], among others. They validated their own proposed PBE submodels with experimental data of pure systems by variation of stirrer speed and for small dispersed phase fractions (0.025<φ<0.15). Another example is the work of Maaß et al. [9], who studied breakage-dominated systems by varying the disperse phase fraction up to 40% (0.05<φ<0.4). With PBE simulations, they could reproduce their experimental results.

In this work, we take a first step towards applying PBEs in particle-stabilized liquid/liquid systems. A coalescence efficiency sub-model is developed that considers the coalescence efficiency reduction due to the adsorption of particles at the liquid/liquid interface. Experimental data of time-resolved drop size distributions of particle-stabilized dispersions are used to elaborate this modified sub-model. In the following, the theoretical PBE framework is introduced, and the desorption energy of particles and the particle coverage degree are defined. Both quantities are linked to the coalescence inhibition by particles and will be considered in the modified coalescence efficiency model.

### 1.1. Population Balance Equations

In PBEs, the change of a drop size distribution (density function of number) *f* over time is formulated with a mass balance that includes terms accounting for the fact that drops can be created or destroyed either by coalescence (${\dot{B}}_{c},{\dot{D}}_{c}$) or by breakage (${\dot{B}}_{b},{\dot{D}}_{b}$):

(1) $$\frac{df(d,t)}{dt}={\dot{B}}_{c}-{\dot{D}}_{c}+{\dot{B}}_{b}-{\dot{D}}_{b}.$$

With the assumption of ideal mixing, spatial dependency of the drop size distribution can be neglected. Birth- and death terms are represented with submodels for coalescence and breakage rates.

The PBE for a discontinuously operated stirred liquid/liquid system is defined by [8]:

(2) df(d,t)dt=12∫0dξ(d′,d″)λ(d′,d″)f(d′,t)f(d″,t)dd′︸B˙c−f(d,t)∫0dmax−dξ(d,d′)λ(d,d′)f(d′,t)dd′︸D˙c+∫ddmaxν(d′)β(d,d′)g(d′)f(d′,t)dd′︸B˙b−g(d)f(d,t)∫abg(d)f(d,t)︸D˙b.

Equation (2) includes the coalescence efficiency λ, drop-drop collision frequency ξ, the breakage rate of a drop *g*, the daughter drop size distribution β and the number of daughter drops per breakage event ν. Here, the drops involved are considered with diameters *d*, $d^{\prime }$, and $d^{\prime \prime }$ depending on whether there is a source or sink in the breakage or coalescence processes.

Depending on the used submodels, the individual terms in Equation (2) include different physical properties of the two phases and system variables, such as the mean energy input ϵ generated by the impeller. In the reviews from Liao and Lucas, several submodels for drop breakage [10] and coalescence rates [11] can be found; the most commonly used are the submodels by C&T [8].

Ribeiro et al. [12], e.g., successfully applied the submodels from C&T to simulate agitated toluene in water dispersions. Moreover, in the aforementioned work from Maaß et al. [9] for breakage-dominated systems, the submodels from C&T were used.

The individual terms of the PBE (Equation (2)) from C&T [8] are presented in detail in the following section.

The product of the coalescence efficiency λ, which represents the probability that two drops merge into one, and the drop-drop collision frequency ξ is defined as the coalescence rate *F*. Therefore, the coalescence rate *F* of two merging drops with the size $d_{1}$ and $d_{2}$ can be described by the following equation:

(3) $$F\left(d_{1},d_{2}\right)=\xi \left(d_{1},d_{2}\right)\cdot \lambda \left(d_{1},d_{2}\right).$$

The collision frequency ξ by C&T [8] includes the assumption that the movement of two colliding drops in a turbulent flow corresponds to the random movement of gas molecules in the classical gas theory. Tsouris [13] extended the collision frequency based on the work of C&T [8] by a damping factor, considering the impact of disperse phase fraction φ. The modified equation, which also considers the energy dissipation rate ϵ, is defined as:

(4) $$\xi \left(d_{1},d_{2}\right)=c_{1,c}\frac{\epsilon^{1/3}}{\left(1+\varphi \right)}{\left(d_{1}+d_{2}\right)}^{2}{\left(d_{1}^{2/3}+d_{2}^{2/3}\right)}^{1/2}.$$

The coalescence efficiency λ, which is based on the film drainage model for an immobile interface [8] is

(5) $$\lambda \left(d_{1},d_{2}\right)=\exp\left[-c_{2,c}\frac{\eta_{c}\rho_{c}\epsilon }{\gamma_{o,w}^{2}{\left(1+\varphi \right)}^{3}}{\left(\frac{d_{1}d_{2}}{d_{1}+d_{2}}\right)}^{4}\right].$$

The coalescence frequency and efficiency are functions of the diameters of the involved drops d’ and d” (in case of birth by coalescence) or d and d’ (in case of death by coalescence), cf. Equation (2). The constants $c_{1,c}$ and $c_{2,c}$ in Equations (4) and (5) are free parameters that can be determined by fitting to experimental data.

C&T [8] assume in their breakage model that drop-eddy collisions are the reason for drop breakage and describe the breakage rate *g* as a function of the mean energy dissipation rate ϵ of the system according to Kolmogorov’s turbulence theory [14] with two further free parameters $c_{1,b}$ and $c_{2,b}$:

(6) $$g\left(d\right)=c_{1,b}\frac{\epsilon^{1/3}}{\left(1+\varphi \right)d^{2/3}}\exp\left(-c_{2,b}\frac{\gamma_{o,w}{\left(1+\varphi \right)}^{2}}{\rho_{d}\epsilon^{2/3}d^{5/3}}\right).$$

The breakage birth rate depends on the daughter drop size distribution β and the number of daughter drops per breakage event ν (cf. Equation (2)). Several models assume a binary breakage (ν=2), which was confirmed in experimental studies on drop breakage [15]. The splitting of the drop volume into daughter drops during a breakage process can be modeled with statistical, phenomenological, or empirical probability density functions [10]. Maaß et al. [16] reported that these daughter drop size distributions (DDSD) could have a significant impact on the shape of the volume-based density drop size distribution $q_{3}$.

For a binary breakage process, normal distributions [8], bimodal distributions [16], as well as beta distributions [17] are often utilized.

In the model of C&T [8], a DDSD is used where the daughter drops $V_{d}$ are normally distributed around the mean value $\mu =\frac{V_{m}}{\nu }$ with $V_{m}$, the volume of the mother drop. The DDSD is defined with the standard deviation $\sigma =\frac{\mu }{C}$ and the dimensionless daughter drop volume $f_{d}=\frac{V_{d}}{V_{m}}$ to:

(7) $$\beta_{norm.}=\frac{1}{\sigma \sqrt{2\pi }}\exp\left(-\frac{{\left(f_{d}-\mu \right)}^{2}}{2\sigma^{2}}\right).$$

The parameter C, in the definition of the standard deviation σ, represents the width of the normal distribution. Another option is the use of a beta distribution, including the gamma function Γ, and the parameters *a* and *b*, which are defined based on previous work [18] to a=b=2. To achieve a symmetrical distribution over $f_{d}$, *a* and *b* must be equal, which is necessary in case of binary breakage [16]:

(8) $$\beta_{beta}=\frac{\Gamma (a+b)}{\Gamma (a)\Gamma (b)}f_{d}^{\left(a-1\right)}{\left(1-f_{d}\right)}^{\left(b-1\right)}.$$

Normal and beta-distributed DDSDs are bell-shaped, so the highest probability occurs for breakage into drops of equal volumes ($f_{d}=0.5$). In contrast, Hesketh et al. [15] and Maaß et al. [16] observed unequal-sized breakup. Based on experimental results on single drop breakage behavior, Maaß et al. [16] proposed a bimodal (m-shaped) distribution:

$$\beta_{bimodal}=\left\{\begin{array}{cc} \frac{0.5}{\sigma \sqrt{2\pi }}\exp\left(-\frac{{\left(f_{d}-\mu \right)}^{2}}{2\sigma^{2}}\right) & f_{d}\leq 0.5\\ \frac{0.5}{\sigma \sqrt{2\pi }}\exp\left(-\frac{{\left(V_{m}-f_{d}-\mu \right)}^{2}}{2\sigma^{2}}\right) & f_{d}>0.5. \end{array}\right.$$

Analogous to the normal distribution, the width of the two distribution peaks can be varied with the parameter *C* implemented in the standard deviation σ.

**Figure 1:**
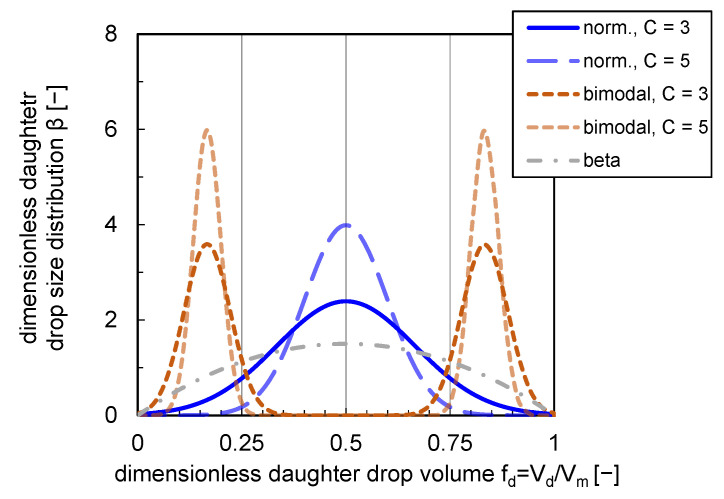
Normal (norm.), bimodal and beta DDSDs. Distributions are plotted with a variety of distribution width (C=3 and C=5) for bimodal and normal distributions.

The shapes of the presented DDSDs are depicted in Figure 1. The impact of a variation of parameter *C* affecting the distribution widths and shape is shown for a normal and bimodal distribution. For the bimodal distribution, the formation probability of two equal-sized daughter drops is zero. The maximum probability is reached at a ratio $f_{d}$ of 1/6 and 5/6 (see Figure 1).

Though it is well established to use statistical DDSD, studies showed that the DDSD, and consequently breakage processes, depend on various parameters. The number of daughter drops and their size distribution differs depending on flow properties, physical properties of the liquid phases, as well as process parameters [16]. In this study, stochastic distributions were used since an exact experimental determination of the DDSD for a stirred system is elaborate or hardly possible.

As a first approach towards modeling particle-stabilized systems, the well-known C&T submodels are used as a basis in this work. A drawback of the submodels from C&T is the missing consideration of the dispersed phase viscosity. Submodels by other authors which take viscous forces in their breakage rate and coalescence efficiency into account, however, contain further free parameters (e.g., [17]).

To account for the impact of nanoparticles on drop size distributions, the focus is on modifying the coalescence efficiency submodel. Although the particles may have an impact on breakage processes as well, it is assumed that the decisive influence of particles on the DSD is the significant reduction of the coalescence rate due to a reduction of the coalescence efficiency caused by the adsorption of the particles at the interface and steric hindrance, respectively.

Since this work focuses on coalescence efficiency modification, the three main model approaches and a recently published approach for this submodel are presented. In the mechanistic model approach, the film drainage model from C&T, it is assumed that coalescence occurs when the contact time exceeds the time required for the thin film of continuous phase between the drops to be drained. In this approach, drop interactions and film drainage are treated as independent phenomena. Moreover, regarding the contact time and drainage time defined by C&T, different descriptions of these characteristic times were proposed by several authors. An overview can be taken from the review of Kamp et al. [19]. The approaches exhibit different dependencies on the physical properties of the phases. As described by Kamp et al. [19], validation of the equations for contact and drainage times would be necessary to describe film drainage, which can be done with laborious single-drop coalescence experiments.

The energy model of Sovova [20] is based on the assumption that for coalescence to occur, the surface energy of the drops must be exceeded by the kinetic energy of the collision. It asserts that, without limitation, increasing relative velocity between the drops leads to a higher coalescence probability. Contrary to the energy model, Lehr et al. [21] experimentally determined a maximum velocity up to which coalescence occurs, but above only a repulsion takes place. Hence, Lehr et al. [21] proposed a semi-empirical approach considering the critical relative collision velocity between drops. A drawback of this approach is that experiments have to be performed to determine critical collision velocities, which is analog to the experimental determination of drainage and contact times, elaborate.

In a recent work from Ozan et al. [22], a new framework combining the three approaches mentioned above is presented. An advantage of their coalescence kernel is that it assumes no independence of the above effects and can also predict repulsion at high collision velocities. The model was validated using data on bubbly pipe flows. The application of this approach would first need to be analyzed for stirred liquid/liquid systems.

The established coalescence efficiency based on the film drainage model of C&T was chosen as a suitable submodel in this work. The definitions of drainage and contact times of C&T were used as a first approach since an experimental validation of the contact and drainage times proposed by other authors through experiments is a major research topic of its own and is not the focus of this work.

### 1.2. Desorption Energy and Particle Coverage Degree

The basic mechanisms of coalescence inhibition by particles are still not completely understood, and the impact of substance variables, such as particle shape [23], and system variables, such as energy input [24] on coalescence still is under investigation.

Particles adsorb nearly irreversibly at the liquid/liquid interface if the free energy needed to detach the particle from the interface is high. In the following, spherical particles are considered, as they were also used in the experiments. The desorption energy depends on the contact angle, the interfacial tension between the liquid phases, and the size of the particles and can be calculated for a spherical particle by [25]:

(9) $$\Delta E=\pi \cdot r_{p}^{2}\cdot \gamma_{o,w}\cdot {\left(1\pm cos\theta \right)}^{2}.$$

High energy values are achieved for particles when the particles are wetted by both phases, i.e., have a contact angle close to $90^{\circ }$.

Arditty et al. [26] state that in the case of particle-stabilized dispersions, coalescence is hindered due to a dense particle film acting as a steric barrier at the interface. This inhibited coalescence depends on the degree of interface covered by particles [7]:

(10) $$\Theta =\frac{A_{p,cov}\cdot n_{p,eff}}{A_{d,tot}\cdot \left(1-\varepsilon \right)}.$$

**Figure 2:**
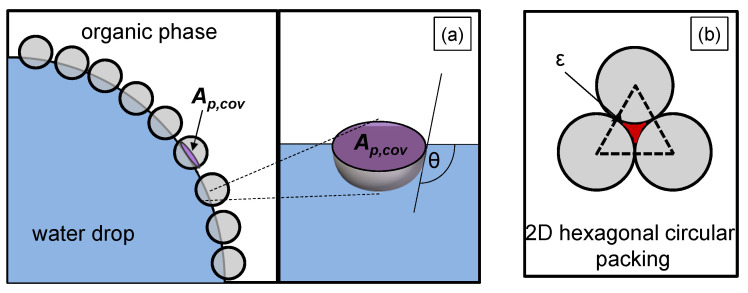
Illustration of (**a**) the planar interface $A_{p,cov}$ of a particle adsorbed at the liquid/liquid interface (purple marked), with a contact angle θ, (**b**) the void fraction ε of a hexagonal two-dimensional (2D) packed spherical particle layer (red marked).

It includes the planar interface of the spherical particle between oil and water $A_{p,cov}$, which is eliminated by the spherical particle resting on the interface. This eliminated area is illustrated in Figure 2a, it depends on the particle radius $r_{p}$ and the contact angle of the particle θ:

(11) $$A_{p,cov}=\pi r_{p}^{2}sin^{2}\theta .$$

$A_{d,tot}$ in Equation (10) represents the total liquid/liquid interface, $n_{p,eff}$ the number of particles that effectively adsorb at the liquid/liquid interface, and ε the void fraction. The void fraction considers that complete coverage of the drop surface cannot be achieved due to the gaps between the particles (red marked in Figure 2b). The minimum void fraction of a hexagonal circular packing, which is illustrated in Figure 2b, corresponds to the void fraction on the drop surface (2D) and is ε=0.093.

Experimental studies from Stock et al. [27] showed that silica spheres with the same modification as in this work built monolayers with a nearly hexagonal, close-packed structure at the interface. For the used particles in this work, a voidage of ε=0.07 was determined, which is close to a 2D-hexagonal lattice [3].

If the amount of particles is insufficient to cover the whole generated interface, drops will coalesce until the interface is completely covered; this time-dependent phenomenon is defined as limited coalescence [26]. If limited coalescence occurs, it depends on factors such as interface generation induced by energy input, the number of adsorbed particles at the interfaces, and the wettability of the particles.

If two coalescing drops are entirely covered with particles, a part of those particles would theoretically have to be detached from the interface so that coalescence into a larger drop with a spherical shape can ensue because coalescence reduces the available liquid/liquid interfacial area for the particles. The energy ΔE (Equation (9)) required to detach these particles, thus, represents an energy barrier against coalescence [25].

Thus, the energy barrier increases rapidly at high particle coverage rates of the interface, so that in this case, drops are long-term stable against coalescence.

Due to particle–particle interactions, the coverage distribution of particles at an interface can be affected. Particles with a neutral or weak charge are more likely to form clusters than particles with a substantial charge and long-range dipole moment, respectively [28]. In calculating the particle coverage degree (Equation (10)), it is assumed that the particles form a hexagonal close-packing monolayer at the interface and do not form agglomerates.

The mobility and deformability of the interface are, in the case of particle-stabilized dispersions, significantly influenced by the interface coverage degree by particles. With increasing particle coverage, the mobility and deformability of the interface are reduced. This affects the film outflow velocities and the film stability during the coalescence process and leads to inhibited coalescence with rising interface coverage degrees by particles.

Apart from the formation of a steric barrier, the particles also influence parameters such as density and viscosity, which in turn also affect the mobility and deformability of the drop surfaces and the film drainage process during coalescence (see Equation (5)). Particle suspensions often show a rising viscosity and exhibit shear thinning and viscoelastic flow behavior compared to pure liquids. This was shown, among others, in the work of Hohl et al. [29] for non-spherical hydrophobized silica particles suspended in 1-dodecene and by Mondragon et al. [30] for aqueous silica suspensions. In the context of the flow behavior of nanoparticle suspensions, particle shape has a significant impact [31]. Non-spherical polydisperse particles can interlock with each other; their degree of interaction is generally greater than that of spherical particles and can lead to an increase in viscosity at low shear rates of up to nearly two orders of magnitude in the same particle concentration range ($w_{p}=0$–1%) as in this work [29]. Particle mass fractions in the relevant range for particle-stabilized liquid/liquid dispersions also lead to a modest increase in the density of the phases [32].

According to Chesters [33], an increase in the viscosity of the continuous phase reduces the mobility of the phase interface. The film flow is slowed down, thus, reducing coalescence. The coalescence can also be diminished by increasing the density of the continuous phase. The density of the dispersed phase influences the inertial force of the drops, which strives to maintain the state of motion of the drops [19]. The change in inertial force causes a change in the kinetic energy of the drops and, thus, a change in the drop-drop collision velocity, which influences the coalescence rate.

Consequently, when predicting the DSD of particle-stabilized liquid/liquid dispersions, it is relevant to consider the impact of the particles on physical properties and particle-linked quantities as interface coverage degree by particles or the desorption energy of particles in the PBEs to model the inhibited coalescence in these systems successfully.

Therefore, common model approaches need to be adapted for particle-stabilized systems [7]. So far, the adaption of PBE submodels has only been discussed or performed for other additive-containing systems, e.g., dispersions stabilized by surfactants. Maindarkar et al. [34] developed PBE submodels for dispersions produced with high-pressure homogenization. In their submodels, the interface coverage degree by surfactant molecules is considered. A modified turbulent breakage frequency in dependency of the homogenizer pressure was developed. The model was validated with experimental data of surfactant stabilized dispersions [34]. Alopaeus et al. extended a high-order moment conserving method for population balances. The impact of surfactants on liquid/liquid dispersions was implemented with a material balance approach. Surfactant concentrations in the continuous phase, dispersed phase, and at the interface were calculated via the mass balances. The impact of the surfactant concentration on drop size distribution could be simulated successfully [35].

These approaches from surfactant systems cannot simply be adopted for particle-stabilized systems due to their different stabilization mechanisms. Adsorption energies are in another order of magnitude so that in contrast to surfactants, particles with a proper wettability can not easily de- and adsorb from an interface. Additionally, the stabilization by surfactants is mainly governed by their high impact on interfacial tension [36].

For particle-stabilized systems, Tsabet and Fradette [7] modified semi-empirical correlations based on the work of Calabrese et al. [37] to model mean drop sizes. They introduced four efficiencies (a)–(d) during particle attachment at a drop interface:

(a) particle/drop collision efficiency, which depends on the film drainage process,
(b) initial attachment efficiency, which considers the impact of the three-phase contact line formation,
(c) particle attachment efficiency, which describes the ability of the particles to remain attached at the interface,
(d) the drop coverage efficiency, which defines the system’s ability to prevent coalescence as a function of particle coverage.

Tsabet and Fradette [7] showed that their modified semi-empirical equation could predict particle-stabilized mean drop sizes in good agreement with experimental results.

It has to be considered that the phenomenon of limited coalescence that occurs in particle-stabilized systems is time-dependent, as explained at the beginning of this section. The consideration of the time-varying degree of coverage by particles during a stirring process cannot be taken into account with the approach of semi-empirical correlations, as no time-resolved DSDs can be calculated. In addition, the semi-empirical correlation approach only provides the mean drop size and not the total DSD.

Standard coalescence efficiency submodels cannot describe the particle-induced reduction of coalescence in agitated liquid/liquid systems. Therefore, a modification of the coalescence efficiency submodel based on film drainage from C&T is performed in this work. Own experimental data of DSDs stabilized by well-defined, monodisperse spherical particles were used. In the developed new coalescence efficiency submodel, the energy barrier of particles against coalescence is considered. This barrier is equal to the desorption energy of the particles and depends on the time-dependent interface coverage rate by particles. With this new approach of a modified coalescence efficiency, the time-resolved influence of particles on complete drop size distributions can be simulated.

## 2. Materials and Methods

In this section, the experimental methods, including used substances and particles, as well as reactor set-up and DSD measurement technique, are introduced. Subsequently, the numerical methods used for solving the PBE and modifying a PBE submodel are presented.

### 2.1. Experimental Investigations Stirred Tank

**Figure 3:**
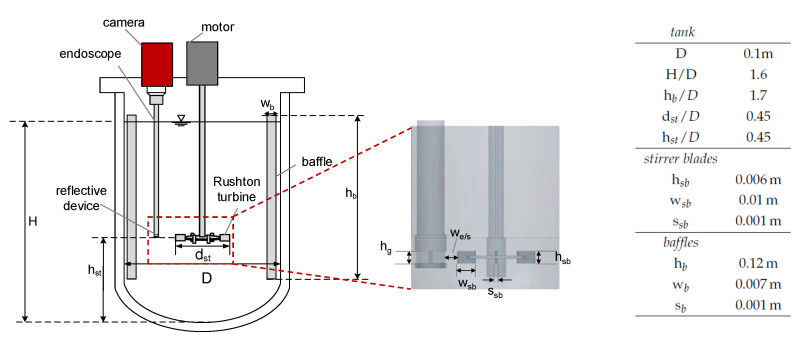
Experimental setup, baffled reactor equipped with a Rushton turbine and in situ endoscope technique and enlarged view of the stirrer and the endoscope with the reflective Teflon device. The corresponding dimensions of the stirred tank set up including baffles (b) and stirrer blades (sb) are shown in the table.

The experiments were performed in a baffled and tempered stirred tank equipped with a Rushton turbine with a total volume of V=700 mL at T=293 K. Figure 3 schematically shows the setup, including the corresponding dimensions. DSDs were measured in situ with an optical endoscope measurement technique (SOPAT GmbH). The endoscope had a length of 40.5 cm, an outer diameter of 21 mm, and a measurement range of 9–1200 μm.

It was connected to a camera (GX 2750, Allied Visions Technology), and a reflective Teflon device was attached to the endoscope tip to improve image quality, with a gap size of $h_{g}\;=\;6\;$ mm (cf. Figure 3). The endoscope tip was positioned in the vicinity of the stirrer blades at a horizontal distance of $w_{e/s}=10\;$ mm to the stirrer blade tips to ensure optimal flow through the gap. A more detailed description of the endoscope measurement method is provided by Maaß et al. [38] and for the Teflon device by Emmerich et al. [39]. Automated image analysis (SOPAT GmbH) was applied to determine the DSD. To ensure accuracy in the determination of the DSD, a sensitivity analysis was performed to verify that enough drops were evaluated at each time step. Consequently, for each mean diameter or DSD, at least 800 drops were recorded and evaluated in a one-time step. The drop size measurements were performed once; the error of endoscope measurements in agitated liquid/liquid systems can be conservatively assumed to be ±20μm [9].

Decane (Roth, purity ≥ 99%) was used as the continuous liquid phase, and ultrapure water (κ = 0.055 μScm${}^{-1}$, Purelab flex 2, Elga) as the dispersed phase. The dispersed volume fraction was φ=0.1 (water in oil). The mass fraction of particles in relation to the mass of the dispersed water phase was varied between $w_{p}=0$–1%. Nearly monodisperse, partially hydrophobic, and positively charged ($\zeta_{Potential}\;=\;56\pm 4$ mV) spherical silica particles were used. The particles had a Sauter mean diameter of $d_{32}=27.6\;\pm \;3\;$ nm, a density of $\rho_{p}=2150\pm 20\;$ kg/m${}^{3}$ and a contact angle of a water drop on particle layer against the air of $\theta \;=\;106\pm 6^{\circ }$, which confirms their hydrophobic character. Simplified and as a first approach, this contact angle is used in this work for the angle of the particles at the liquid/liquid interface, as it is challenging to determine this contact angle reliably. Transmission Electron Microscopy (TEM) pictures of the spherical particles and more detailed information concerning the particles’ modification, properties, and their determination are provided in [3,27]. The following measurement techniques were used to determine the influence of the particles on the physical properties of the two phases. In each case, three replicate measurements were carried out. With a tempered pendant drop method (Dataphysics OCA 15), the interfacial tension was measured in a steady state. The densities of the pure liquid phases were determined with a tempered oscillating U-tube principle (Anton-Paar DA5000M). Particle suspensions were prepared with an ultrasonication homogenizer (Bandelin Sonoplus HD70, P = 70 W, f = 20 kHz, 75% Amplitude); in each case, V=50 mL of suspension were sonicated for 5 min. The rheological behavior of the suspensions were analyzed with a tempered rotational rheometer using a cone and plate system (MCR 302, Measurement system CP60-1, Anton Paar): cone diameter 59.978 mm, angle $1.008^{\circ }$, gap size 0.117μm. Flow curves were measured in a shear rate range of 1–1000 $s^{-1}$ with a measuring point time duration adapted to the shear rate, which was always chosen higher than the reciprocal value of the shear rate. Experimental torque measurements were used to determine the power input. In this work the stirrer speed was varied between *n* = 700–900 rpm, which corresponds to mean energy dissipation rates of $\epsilon_{700rpm}\;=\;0.88\;$W/kg, $\epsilon_{800rpm}\;=\;1.31\;$W/kg and $\epsilon_{900rpm}\;=\;1.87\;$W/kg. The physical properties of the particle-free system ($w_{p}=0\%$) were also determined. The interfacial tension between water and decane is $\gamma_{o,w}=52.13\pm 0.4$ mN/m, the density and dynamic viscosity of the continuous phase: $\rho_{c}=729.9\pm 0.1$ kg/m${}^{3}$ and $\eta_{c}=0.91\pm 0.09$ m Pas. As well as the density and viscosity of the dispersed phase: $\rho_{d}=998.2\pm 0.05$ kg/m${}^{3}$ and $\eta_{d}=1\pm 0.1$ m Pas. All measurements presented were carried out at T=293±0.2 K.

### 2.2. Numerical Investigations

The population balance is an integrodifferential equation that must be solved numerically. The software Parsival^®^ version 7.6a [40] was used for the solution, which solves the coupled mass balance with an adaptive discretization at each time step using the Galerkin-h-p algorithm. With the implemented models for drop breakage and coalescence processes in the software environment, the free model parameters ($c_{1,b}$, $c_{2,b}$, $c_{1,c}$, and $c_{2,c}$) can be fitted to the experimental data with an implemented parameter estimation routine. Initial values for the four fitting parameters and an initial DSD must be specified. With a Dirichlet boundary condition, the value of the initial DSD for $d_{min}$ is defined: $f(d_{min})=0$. The numerical definition limit of the considered DSD was set to the minimum value of $d_{min}=1\;\mu$m and the maximum value of $d_{max}=1000\;\mu$m. Analogous to the DDSD (Equation (7)), a Gaussian normal distribution with a mean of μ=500μm and a standard deviation of σ=25μm was chosen to represent the initial numerical DSD f(d,t=0s).

The optimization algorithm to determine the free model parameters used in Parsival^®^ attempts to minimize the residual between experimental and simulated data using the relative root-mean-square deviation (RRMSD). In this work, the RRMSD was additionally used to indicate the deviations between the results of simulations and experiments.

**Figure 4:**
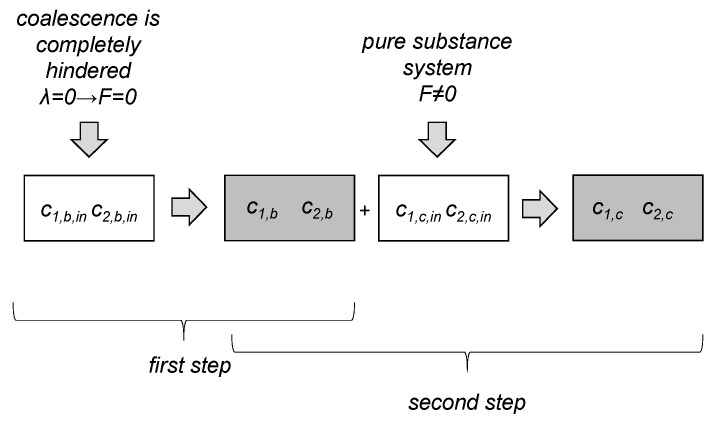
Schematic visualization of the two-step parameter estimation procedure. Initial parameters ($c_{1,b,in}$, $c_{2,b,in}$ for breakage, $c_{1,c,in}$ and $c_{2,c,in}$ for coalescence) were taken from another substance system. In the first step, the initial breakage parameters were fitted to experimental data where coalescence is completely halted (*F* = 0), resulting in two new breakage parameters $c_{1,b}$ and $c_{2,b}$. In a second step, these are kept constant, and the initial free parameters of coalescence were fitted to experimental data where coalescence and breakage occur (pure system), resulting in new coalescence parameters $c_{1,c}$ and $c_{2,c}$.

Simulations were performed with a single zone PBE, with a mean averaged energy input for the whole vessel. The parameter estimation was done in a two-step fit procedure for the four free fitting parameters: $c_{1,b}$, $c_{2,b}$, $c_{1,c}$, and $c_{2,c}$. In the first step, experimental data were used, where the coalescence was completely hindered and only drop breakage occurred to determine $c_{1,b}$ and $c_{2,b}$. These constants were then used for a system where coalescence is nearly uninhibited (pure substance system, $w_{p}=0\%$) to fit $c_{1,c}$ and $c_{2,c}$. Figure 4 shows a schema of the two-step fit procedure. The initial values for the free parameters are listed together with the fitted values in Section 3.2.2. The initial values are based on those obtained in our own works for a pure toluene/water system.

## 3. Results and Discussion

This section starts with the experimental results, and building upon this, the modeling approach and simulation results are shown and evaluated.

### 3.1. Experimental Results

The experimental results concerning the impact of particles on the physical properties such as density, dynamic viscosity, and interfacial tension are presented first. Experimentally determined DSDs for various particle mass fractions are shown subsequently, including their response to a step change in energy input.

#### 3.1.1. Impact of Nanoparticles on Physical Properties

The densities of the particle-stabilized systems were calculated with the mixing theory for binary systems assuming no excess volume. This assumption was proven with a conical Hubbard pycnometer, the procedure and used equation to calculate the density can be taken from the previous work [41]. Despite their partially hydrophobic property, the particles were assigned to the dispersed phase water to determine density, as they adsorb at the interface.

The change in density at T=293 K in the investigated particle mass fraction range is small; a maximum percentage increase of 0.56% occurs at the highest particle mass fraction ($w_{p}=1\%$) in the system, which corresponds to a disperse phase density of $\rho_{d}\left(w_{p}=1\%\right)=1003.88$ kg/m${}^{3}$.

In accordance with previous work [42], the change of interfacial tension in the presence of nanoparticles at the interface is negligible in comparison to the nanoparticle-free system. A maximum deviation of $\Delta \gamma_{o,w}=\pm 1.2$ mN/m occurred compared to the pure system in steady state for particle mass fractions $w_{p}\;=\;0.25$–1% measured at T=293 K. Thus, the interfacial tension $\gamma_{o,w}$ was kept constant using the value of the pure system ($\gamma_{o,w}=52.13\;$ mN/m) for all PBE simulations.

**Figure 5:**
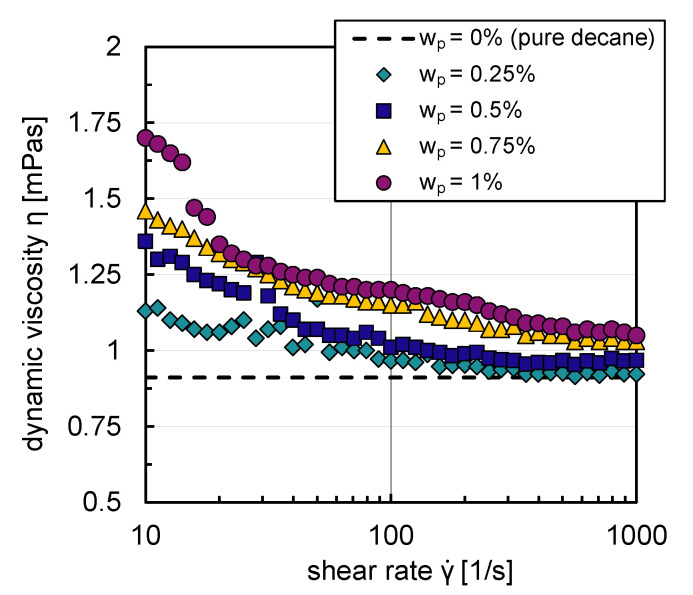
Suspension viscosities with decane as base fluid as a function of shear rate, with different particle mass fractions $w_{p}\;=\;0.25$–1%.

Due to their hydrophobic character, particles do not build stable suspensions in an aqueous environment. The influence of the particles on the rheological flow behavior could, therefore, only be analyzed with decane as the base fluid. Figure 5 shows that decane suspensions exhibit shear thinning flow behavior for all particle mass fractions ($w_{p}=0.25$–1%). For graph clarity, the relative error, calculated from the standard deviation of three replicate measurements of dynamic viscosity, is not shown in Figure 5; it was a maximum of ±7% in the shear rate range studied ($\dot{\gamma }=10$–$1000\;s^{-1}$). An increase of suspension viscosity with rising particle mass fraction can be observed from Figure 5. A maximum increase of Δη=0.8m Pas in suspension viscosity in comparison to the pure system occurred for the lowest shear rate and highest particle mass fraction investigated. The particles only have a very modest influence on the flow behavior; they build weak agglomerate networks, which are broken up with increasing shear rates.

The viscosity deviations compared to the pure system are more significant for small shear rates ($\dot{\gamma }=10$–$100\;s^{-1}$) in contrast to the higher shear rate region ($\dot{\gamma }\;=\;100$–$1000\;s^{-1}$). Low shear rates are present in the reactor’s bulk phase in the investigated energy input range where coalescence processes mainly occur. Higher shear rates exist in the stirrer vicinity, where breakage processes dominate [32].

Since the particles in this work lead to a maximum increase in dynamic viscosity of Δη<1m Pas, the influence is neglected in the first approach in the simulations.

Since the exact shear rates of the locations in the reactor where coalescence and breakage occur can not be considered by using a mean energy input and no division of the reactor into different segments. The shear thinning flow behavior of the suspensions indicates that for the coalescence rather than the breakage processes and, therefore, submodels in PBE, the increased viscosity due to the particles could affect the film drainage process and, thus, on the resulting drop sizes.

#### 3.1.2. Experimental Drop Size Distributions

**Figure 6:**
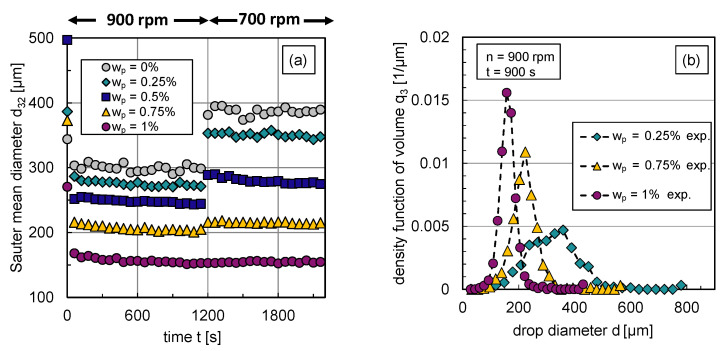
Experimental results: (**a**) Transient Sauter mean diameter with a step-wise change in stirrer speed from n=900rpm to n=700rpm at t=1160 s, (**b**) stationary density-volume distributions $q_{3}$ at n=900rpm for particle mass fractions $w_{p}=0.25\%$, $w_{p}=0.75\%$ and $w_{p}=1\%$.

The impact of the particle mass fraction on the transient Sauter mean diameter $d_{32}$ and corresponding exemplary DSDs $q_{3}$ in steady state are illustrated in Figure 6. Using an abrupt change of agitation speed after a stationary state is reached, the transient response of the Sauter mean diameter was tracked over time. For graphic clarity, the error bars of the experimental data are not illustrated; the error range can be taken from Section 2.

The reduced Sauter mean diameters with rising particle mass fraction shown in Figure 6a indicate that the particles hinder coalescence more efficiently with increasing particle mass fraction respective interface coverage degree. Correspondingly, Figure 6b shows the volume-based DSDs in steady state (t=900 s) at n=900rpm for different particle mass fractions. For graph clarity, only distributions of three representative particle mass fractions are shown. The inhibited coalescence by particles is also visible in the distributions, $d_{max}$ is shifted to lower values with increasing interface coverage degree or particle mass fraction, respectively. The shape of the distribution becomes narrower with increasing particle content. This can also be seen in the associated volume-based $span_{3}$, which is defined by the difference between the 90% and 10% percentile, normalized by the 50% percentile of the distribution. The $span_{3}$ decreases for n=900rpm from 0.8 ($w_{p}=0.25\%$) to 0.49 ($w_{p}=1\%$).

The increase in the Sauter mean diameter after the reduction of the energy dissipation rate ϵ, carried out by reducing the stirrer speed *n*, also shows how particles affect the balance between drop breakage and coalescence. For particle mass fractions $w_{p}=0.25$–0.75%, the particle coverage degree is too low to fully cover the generated liquid/liquid interface at n=900rpm. Therefore, coalescence occurs after a reduction of energy input, which increases the Sauter mean diameter.

As the interface coverage degree Θ increases, coalescence is increasingly inhibited, which is evident from a smaller increase in $d_{32}$ after energy input reduction with rising particle mass fraction $w_{p}$ (see Figure 6a). Thus, particles adsorb at the phase interface and hinder coalescence. Figure 6a also shows that steady-state values of the Sauter mean diameter are reached quickly in all cases and at a slightly later point in time as the particle mass fraction increases. Thus, it takes longer to achieve equilibrium between drop breakage and coalescence if coalescence is inhibited. The equilibrium between drop breakage and coalescence in steady state shifts with increasing interface coverage degree evermore towards breakage until coalescence is completely halted (F=0) at a particle mass fraction of $w_{p}=1\%$; no increase in the Sauter mean diameter occurs after decreasing the energy dissipation rate in this case.

Using the experimental data from Figure 6a, the breakage parameters $c_{1,b}$, $c_{2,b}$ and coalescence parameters $c_{1,c}$ and $c_{2,c}$ can be determined. The transient Sauter mean diameter curves for $w_{p}=1\%$ at n=900rpm are suitable for the first step of the parameter estimation routine already discussed in Figure 4; for the second step, the data of the particle-free system ($w_{p}=0\%$) is used.

### 3.2. Numerical Results

This section presents the parameter estimation followed by the developed modified coalescence efficiency model and finally the modeling results compared with experimental data.

#### 3.2.1. Parameter Estimation

The obtained fitted parameters are compiled in Table 1. By use of the transient $w_{p}=1\%$ curve at n=900rpm, for the determination of $c_{1,b}$ and $c_{2,b}$, the mean RRMSD between fitted and experimental values was $\bar{r}=5.29\%$. The determination of $c_{1,c}$ and $c_{2,c}$ by use of the $w_{p}=0\%$ curve at n=900rpm exhibit a RRMSD of $\bar{r}=3.72\%$. In both cases, the fits were performed with a normally distributed DDSD with C=3.

#### 3.2.2. Development of Modified Coalescence Efficiency Model

The change of the physical properties density and viscosity due to the presence of nanoparticles was taken into account in the submodels by C&T. Changing only these physical parameters, however, does not suffice to predict the reduction of the Sauter mean diameter by particles shown in Figure 6. For example, the simulation with adapted physical properties for $w_{p}=1\%$ using the original C&T submodels and the free parameters shown in Table 1 leads to a Sauter mean diameter of $d_{32}=275\;\mu$m in steady state for n=900rpm, with a conservative assumption that an increase in continuous viscosity of 1.72m Pas ($\dot{\gamma }=10\;s^{-1}$) occurred (see Section 3.1.1). This shows a reduction of the Sauter diameter of only 30μm in comparison to the pure system ($w_{p}=0\%$). Experiments, however, resulted in a Sauter mean diameter of $d_{32}=150\;\mu$m (see Figure 6) for $w_{p}=1\%$ and a reduction of $\Delta d_{32}=155\;\mu$m compared to the pure system. This comparison shows a deviation between experiments and simulation of at least 83% for $w_{p}=1\%$ at n=900rpm. Thus, the original model of C&T cannot represent the influence of nanoparticles via the changed physical quantities of density and viscosity. A simulation with adapted physical properties for $w_{p}=1\%$ using the original C&T submodels and the fit parameters shown in Table 1 in comparison to the experimental results can be taken from the Supplementary Materials (Figure S1).

Therefore, the submodels need to be further developed to account for the presence of nanoparticles at the interface. The modified submodel for coalescence efficiency is investigated as a first approach in the following. As explained in Section 3.1.1, the viscosities of the pure systems were used for all other simulations.

The effect of particles on coalescence efficiency is analyzed by introducing an additional parameter $c_{np,c}$ into the coalescence efficiency. Like the original coalescence efficiency of C&T (Equation (5)), the modified equation should also assume a range of values between zero and one. The use of nanoparticles should have a damping effect on the coalescence efficiency. At the same time, the modified coalescence efficiency for particle-free systems should be in agreement with the original one of C&T (Equation (5)). This is achieved if the parameter is multiplied in the exponential term and a value of $c_{np,c}=1$ is used for an interface coverage degree of zero (a pure liquid/liquid system, respectively). The coalescence efficiency equation modified by the parameter $c_{np,c}$ is given by

(12) $$\lambda \left(d_{1},d_{2}\right)=exp\left[-c_{np,c}c_{2,c}\frac{\eta_{c}\rho_{c}\epsilon }{\gamma_{o,w}^{2}{\left(1+\varphi \right)}^{3}}{\left(\frac{d_{1}d_{2}}{d_{1}+d_{2}}\right)}^{4}\right].$$

The free parameter $c_{np,c}$ is fitted to the particle-stabilized experimental results with the obtained parameter set, already shown in Table 1. The maximum RRMSD by fitting $c_{np,c}$ to the transient DSD for each particle mass fraction was $\bar{r}=7.21\%$. With the experimental transient DSD results (see Figure 6), the interface coverage degree (Equation (10)) for each investigated particle mass fraction was calculated.

From the experimental results presented in Figure 6a, it can be concluded that at a particle mass fraction of $w_{p}=1\%$ coalescence is completely hindered at n=900 rpm in steady state. With the assumption that the drops are fully covered by particles (Θ=1) in this case, the effective number of particles $n_{p,eff}$ positioned at the interface can be calculated. The ratio between the number of particles available in the system and the number of particles at the interface is also used to calculate the interface coverage degree Θ for lower particle mass fractions at n=900 rpm.

**Figure 7:**
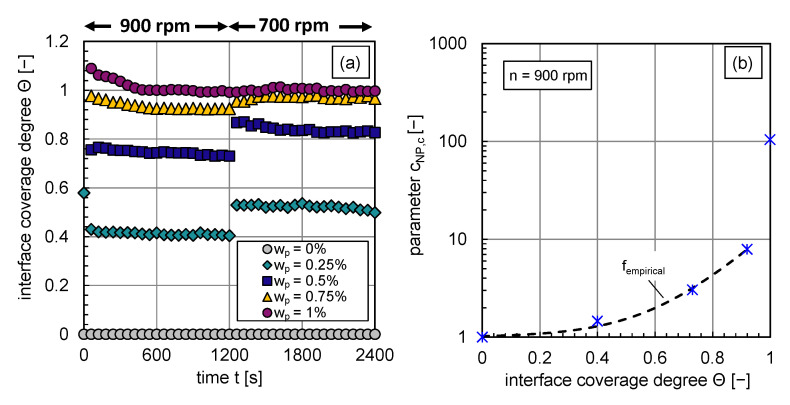
(**a**) Transient calculated interface coverage degree for $w_{p}=0$–1% before and after an abrupt stirrer speed decrease at t = 1160 s, (**b**) fitted parameter $c_{np,c}$ as a function of the coverage degree Θ by particles at n=900rpm (blue stars), fitted curve (black dotted line) $f_{empricial}$ for interface coverage degrees lower 0.9.

The calculated interface coverage degree curves for each investigated particle mass fraction are shown in Figure 7a. The dependency of the coverage degree on the energy input is clearly visible. An abrupt decrease in energy input leads to bigger drop sizes, a decrease in available interfacial area, and an increase in the interface coverage degree as long as coalescence is not completely inhibited. The objective is to correlate, as a first step, the change in coalescence efficiency, expressed via $c_{np,c}$, with the interface coverage degree at n=900rpm. So that the inhibited coalescence, represented by the decrease of the coalescence efficiency λ or increase in the parameter $c_{np,c}$ (see also Equation (12)), is expressed by the increase of the particle coverage degree at the phase interface.

The functional empirical relationship between the parameter $c_{np,c}$ and the interface coverage degree Θ is plotted in Figure 7b. It can be described with the function $f_{empirical}$ indicated by the black dashed line (cf. Figure 7b) for interface coverage degrees until Θ≤0.9:

(13) $$f_{empirical}=1.16\cdot 10^{-2}\cdot exp\left(7.7\cdot \Theta \right)+1$$

with a coefficient of determination of $R^{2}=0.992$. The calculation of $c_{np,c}$ for higher interface coverage degrees by particles is explained in the following paragraph.

As a next step, to describe the coalescence inhibition of drops that are almost entirely covered with particles, an approach is chosen where the energy barrier associated with the energy needed to desorb a particle is taken into account based on [25]. In Section 1.2 the energy barrier against coalescence was introduced; in this section, the concrete implementation of the energy barrier in the coalescence efficiency is presented.

During the coalescence of two drops, the total interfacial area is reduced. If the particle-covered interface of the individual drops is larger than the interface created after coalescence; particles theoretically need to desorb from the interface for a successful coalescence with subsequent shape relaxation into a spherical shape. The energy required to desorb the corresponding number of particles is the energy barrier against coalescence $E_{barrier}$ and depends on the calculated number of particles $n_{d}$ that have to desorb and the desorption energy of one particle ΔE (Equation (9)):

(14) $$E_{barrier}=n_{d}\cdot \Delta E.$$

**Figure 8:**
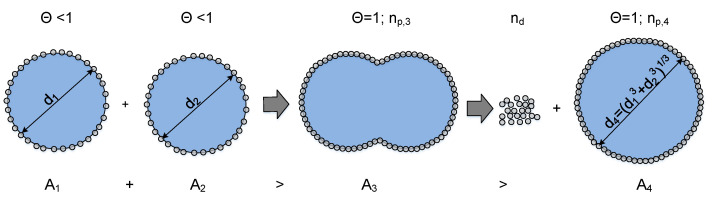
Number of particles $n_{d}$ that theoretically need to be removed from the interface during the coalescence process of two drops that are covered with particles. The energy needed to desorb particles from the interface is equal to an energy barrier against coalescence, based on [25].

A schematic representation of a coalescence process of two drops, where particles have to be desorbed, is depicted in Figure 8.

The number of particles that need theoretically to desorb is given by the quotient between the reduced surface area ($A_{3}-A_{4}$) and the sum of the eliminated area by the particles $A_{p,cov}$ (Equation (11)) and the free surface around the particle $A_{p,gap}$:

(15) $$n_{d}=n_{p,3}-n_{p,4}=\frac{A_{3}-A_{4}}{A_{p,cov}+A_{p,gap}}.$$

In Equation (15), $A_{p,gap}$ can be defined via the voidage ε:

(16) $$A_{p,gap}=A_{p,cov}\cdot \frac{\varepsilon }{1-\varepsilon }.$$

The equation to calculate the number of particles which need to be desorbed results in

(17) $$n_{d}=n_{p,3}-n_{p,4}=\frac{A_{3}-A_{4}}{A_{p,cov}}\cdot \left(1-\varepsilon \right).$$

In the following, two merging drops with equal coverage degrees are considered. The minimum surface area $A_{3}$ required for this case so that all particles can attach as a monolayer during coalescence can be calculated by

(18) $$A_{3}=\Theta \cdot \left(A_{1}+A_{2}\right)$$

and the surface of the coalesced drop in a spherical shape by

(19) $$A_{4}={\left(A_{1}^{3/2}+A_{2}^{3/2}\right)}^{2/3}.$$

Implementing the presented equations into the solver Parsival^®^, it is possible to calculate the number of particles $n_{d}$ that theoretically must be removed at each time step of a simulation.

This approach depends on the interface coverage degree and is only considered if the interface is completely filled with particles during or before coalescence.

The coverage rate determining if the approach needs to be taken into account, considering that the volume of the dispersed water is conserved, can be determined via

(20) $$\Theta >\frac{{\left(A_{1}^{3/2}+A_{2}^{3/2}\right)}^{2/3}}{A_{1}+A_{2}}.$$

The lowest coverage degree where particles need to desorb for coalescence to occur is reached if the colliding drops have the same size $\left(A_{1}\;=\;A_{2}\right)$. Therefore, the minimum possible coverage value for two colliding drops with an equal coverage degree and size is Θ=0.79. Above a coverage degree of Θ=1, coalescence is inhibited for all drops of the system.

Using the empirical modeling approach for low interface coverage degrees and considering the energy barrier at high interface coverage degrees, the following case distinction can be used to define the parameter $c_{np,c}$ in the coalescence efficiency with the substance-specific dimensional fit parameter $c_{des}\left[J^{-1}\right]$:

$$c_{np,c}=\left\{\begin{array}{cc} f_{emprirical} & 0\leq \Theta <0.79\\ c_{des}\cdot E_{barrier} & \Theta \geq 0.79 \end{array}\right.$$

For case discrimination, the interface coverage degree by particles Θ is calculated at each time step in Parsival^®^ and via the case discrimination, $c_{np,c}$ is determined. With experimental data for Θ≥0.79, the new fit parameter $c_{des}\;=\;6.17\cdot 10^{10}J^{-1}$ with an RRMSD of $\bar{r}=5.16\%$ of the implemented, modified coalescence efficiency equation was determined using the parameter estimation routine of Parsival^®^.

### 3.3. Comparison of Simulated and Experimental Results

In this section, drop size distributions (DSDs) and mean diameters in transient and steady-state are modeled and compared with experimental results. The impact of interface coverage degree by particles, energy input, and the choice of daughter drop size distribution (DDSD) is analyzed. A normally distributed DDSD with C=3, which gave the best simulation results for the distributions, was used for all simulations performed. The influence of the DDSD shape on the DSD is discussed in more detail in Section 3.3.3.

#### 3.3.1. Comparison of Simulated and Experimental Transient Sauter Mean Diameters

**Figure 9:**
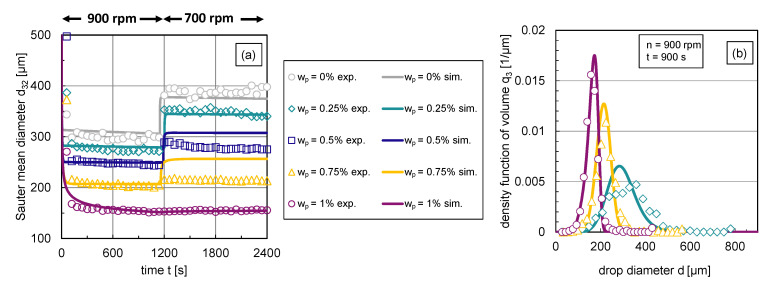
Comparison of experimental and simulation results: Simulations (lines) with the use of the free parameters listed in Table 1 and modified coalescence efficiency (Equation (12)) and experimental data (symbols). (**a**) Transient Sauter mean diameter with a step-wise change in stirrer speed from n=900rpm to n=700rpm at t=1160s, (**b**) stationary density-volume distributions $q_{3}$ at n=900rpm for particle mass fractions $w_{p}=0.25\%,w_{p}=0.75\%$ and $w_{p}=1\%$.

A comparison of transient experimental and simulated Sauter mean diameter curves is shown in Figure 9a. In the modified coalescence efficiency model, the interface coverage degree is calculated dynamically through the presented case distinction, for example, after transient changes of drop size resulting from changes in energy dissipation rate.

For low interface coverage degrees ($w_{p}=0.25\%$), the increase in Sauter mean diameter can be accurately described. The reduced coalescence is slightly underestimated for higher particle mass fractions ($w_{p}=0.5$–0.75%), showing that the shift between breakage and coalescence towards breakage is not yet perfectly described. In Figure 9b, the corresponding simulated volume-based density distributions at t=900 s (n=900rpm) are illustrated along with the experimental distributions. The simulated distributions in Figure 9b show only minor deviations from the experimental distributions. The simulated distribution shape corresponds to the experimental one. The modal value is slightly overestimated for all three particle mass fractions in the simulations. The deviations between simulated and experimental distributions decrease with increasing particle mass fraction, whereas the experimental distributions show a smoother course even with higher particle mass fractions. Considering that during the fitting procedure, the free parameters were fitted only to the Sauter mean diameters, the simulated distributions with a normally distributed DDSD with C=3 provide very satisfactory results.

#### 3.3.2. Simulated and Experimental Sauter Mean Diameters and DSDs in Steady State

**Figure 10:**
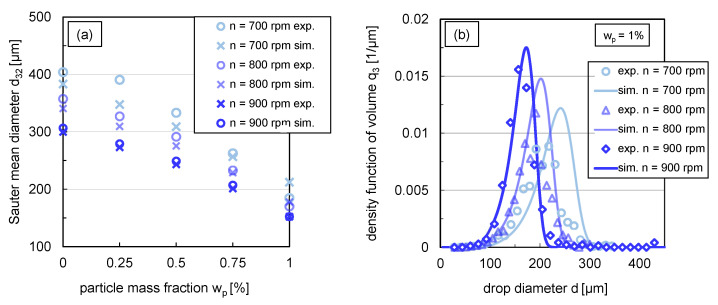
(**a**) Comparison between experimental (circles) and simulated (crosses) Sauter mean diameter for different stirrer speeds (n=700,800,900rpm) in steady state (t=900 s), (**b**) simulations (lines) in comparison with experimental data (symbols) of the volume based density function $q_{3}$ in steady state (t=900 s) for $w_{p}=1\%$ with a variation of stirrer speed. Simulations were performed with free parameters summarised in Table 1 and modified coalescence efficiency according to (Equation (12)).

Figure 10a compares the steady-state simulation results of the Sauter mean diameters as a function of particle mass fraction for different stirrer speeds with experimental data. The steady-state experimental values of the Sauter mean diameters were obtained from the transient curves recorded only at the respective stirrer speed. The experimental steady-state Sauter mean diameters at 700rpm in Figure 10, therefore, differ from the experimental results obtained from the step-wise change from 900 to 700rpm illustrated in Figure 9.

The experimental distributions shown in Figure 6b correspond to the experimental steady-state Sauter mean diameters at a stirrer speed of n=900rpm shown in Figure 10a.

For the systems where coalescence is nearly or completely halted ($w_{p}\geq 0.75\%$), the simulation results show good agreement with experimental data for all energy inputs. There are slightly greater deviations for the lower particle mass fractions till $w_{p}=0.5\%$ at stirrer speeds of 700rpm and 800rpm. This can also be seen in the respective relative root-mean-square deviation (RRMSD) between experimental and simulated Sauter mean diameters summarised in Table 2, which is up to 15% for one experimental condition. In this context, it is important to note that the experimental Sauter mean diameters also have an error of up to ±20μm, which corresponds to a deviation of 5–15%. Consequently, the influence of the energy input on the Sauter mean diameter can be predicted sufficiently precisely with the submodels used.

The impact of energy input on the distribution shape was exemplarily investigated for $w_{p}=1\%$. A comparison of the volume density distribution $q_{3}$ for stirrer speeds between n=700–900rpm from simulations and experiments is shown in Figure 10b. The simulated distributions match the experimental distributions closely. For the stirrer speeds n=700rpm and n=800rpm, the simulated distribution curve of the smallest drop fraction, till around the 40% percentile, is very similar to the experimental distribution shape. Slightly larger deviations exist between the experimental and simulated distribution for the drop fraction above the 50% percentile. The modal value of the distributions is slightly overestimated compared to the experimental results for all three stirrer speeds. Nevertheless, the results of the simulations predict the experimental distributions adequately.

#### 3.3.3. Daughter Drop Size Distribution

**Figure 11:**
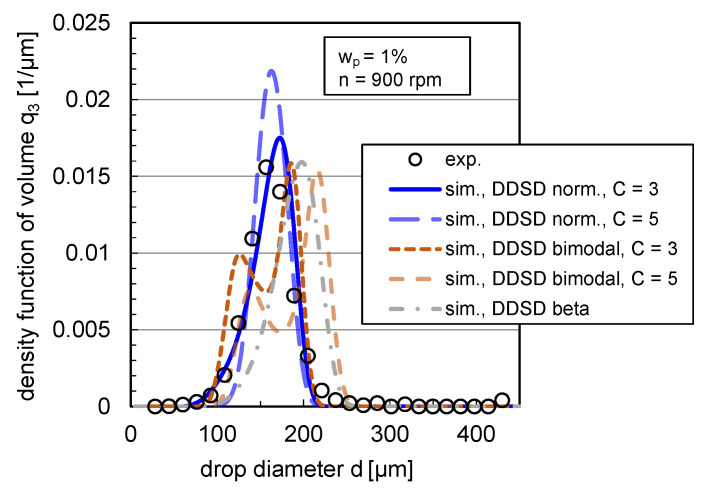
Impact of DDSD shape (normal, bimodal and beta distributed) and width (C=3 and C=5) on volume-based density DSD $q_{3}$ for the case of binary breakage (ν=2). Simulations were performed with the free parameters listed in Table 1 and with the modified coalescence efficiency (Equation (12)) for $w_{p}=1\%$ and n=900rpm in comparison with the experimental distribution. Experimental and simulation results are shown in steady state (t=900 s).

The impact of DDSD type and width on the density distribution of volume $q_{3}$ is depicted exemplarily in steady state for the coalescence-inhibited system ($w_{p}\;=\;1\%$) at n=900rpm in Figure 11.

In agreement with the statement of Maaß et al. [16], the chosen statistical DDSDs have a clear impact on the simulated DSD and lead to different distribution shapes. A bimodal DDSD results for both simulated distribution widths to a bimodal DSD, which does not correspond to the experimental distribution shape (see Figure 11). Using a beta DDSD, the simulated distribution is skewed toward high drop diameters compared to the experimental distribution, overestimating the 10% and 90% percentiles and slightly underestimating the modal value. The normal distribution with a value for the distribution width of C=5 overestimates the modal value; with a width of C=3, the experimental and simulated distributions match accurately. The experimental distributions can, thus, best be reproduced with a drop breakage mechanism, where the probability that the drop breaks into two equal-sized drops is highest instead of breaking into different-sized daughter drops (bimodal, m-shaped DDSD).

## 4. Conclusions and Outlook

Transient and steady-state drop size distributions of a particle-stabilized liquid/liquid system were investigated experimentally using an in situ measurement technique in a stirred tank. The experiments show the impact of particle interface coverage degree on coalescence behavior, for example, after stepwise changes in energy input and tracking of the dynamic changes in Sauter mean diameter for different particle mass fractions. Based on the experiments, a modified PBE submodel to describe the impact of particles with precisely determinable dimensions on the coalescence efficiency was developed. The model can be used to calculate the time-dependent behavior of mean diameters and distributions for different particle interface coverage degrees in good agreement with experimental data. The developed model uses a case distinction depending on the interface coverage degree by particles, which is calculated for each time step of the transient DSD. In the calculation of the interface coverage degree, the impact of contact angle, particle size, particle mass fraction, and energy input is considered. A validation between experimental and simulated values by variation of the energy input and particle mass fraction in steady state was performed. It showed that the changed equilibrium between coalescence and breakage leads to slight deviations of the RRMSD at higher energy inputs and higher deviations at lower energy inputs. The model, thus, underestimates the shift with decreasing energy dissipation rate towards coalescence. Moreover, a DDSD was found to simulate the distribution density function well to match the corresponding experimental distribution almost exactly.

Since this work provides a first approach toward modeling particle-stabilized dispersions via PBEs, there naturally are various options to optimize and refine the submodels further. Additional experimental data could be used in the future, including experiments with particles of different sizes or contact angles and an exact determination of the DDSD. How strongly viscoelastic phenomena caused by the particles also influence coalescence and whether this should also be taken into account in the PBE submodels is an open question. For this purpose, the complex flow behavior of particle-stabilized systems and their eventual impact on DSD should be analyzed in more detail. In the context of complex rheological phenomena, a suitable approach might be to use a multi-zone PBE that accounts for the different local shear rates and, thus, viscosities and moduli or to use CFD simulations for this specific purpose. For lower interface coverage rates, the empirical approach should be modified to describe the reduced coalescence efficiency via physical quantities, such as the increased interface elasticity by particles.

## Figures and Tables

**Table 1:** Initial and fitted parameters for the C&T PBE submodels, using the two-step parameter estimation routine illustrated in Figure 4.

	$c_{1,b}$	$c_{2,b}$	$c_{1,c}$	$c_{2,c}$
initial values	$6.012\cdot 10^{-2}$	$2.833\cdot 10^{-2}$	$1.060\cdot 10^{-4}$	$1.435\cdot 10^{11}$
fitted values	$2.639\cdot 10^{-2}$	$1.218\cdot 10^{-1}$	$3.394\cdot 10^{-1}$	$4.245\cdot 10^{13}$

**Table 2:** Relative root-mean-square deviations in percent between experimental and simulated steady-state Sauter mean diameters, by variation of stirrer speed *n* and particle mass fraction $w_{p}$. For each stirrer speed, the total RRMSD for all 5 particle mass fractions is also tabled.

Particle Mass Fraction $w_{p}$ [%]	0	0.25	0.5	0.75	1	Total
RRMSD ($d_{32,stat}$) [%] *n* = 900 rpm	2.24	2.31	2.41	2.96	0.03	**2.23**
RRMSD ($d_{32,stat}$) [%] *n* = 800 rpm	4.70	5.37	5.49	1.68	4.61	**4.59**
RRMSD ($d_{32,stat}$) [%] *n* = 700 rpm	5.14	11.05	7.29	2.28	15.02	**9.30**

## Data Availability

The data presented in this study are available on request from the corresponding author.

## Supplementary Materials

The following supporting information can be downloaded at: https://www.mdpi.com/article/10.3390/nano13040698/s1, Figure S1: Comparison experimental and simulated transient Sauter mean diameters for $w_{p}=1\%$. Simulations were performed with the original (unmodified) C&T submodels.

## Abbreviations

The following abbreviations are used in this manuscript:

C&T	work from Coulaloglou and Tavlarides [8]
DSD	drop size distribution
DDSD	daughter drop size distribution
PBE	population balance equation
RRMSD	relative root-mean-square deviation
TEM	transmission electron microscopy
w/o	water-in-oil
2D	two-dimensional
**Symbols Used**
*A*	$\left[m^{2}\right]$	area
*a*	[-]	constant in beta distribution
$\dot{B}$	$\left[m^{-4}s^{-1}\right]$	birthrate in PBE
*b*	[-]	constant in beta distribution
*C*	[-]	measure of the width of the normal distribution
*c*	[-]	fitparameter in PBE submodels
$c_{np,c}$	[-]	dimensionless damping factor in coalescence efficiency
$c_{des}$	$\left[J^{-1}\right]$	fitparameter in the energybarrier modell
*d*	[m]	drop diameter (d${}^{\prime }$ and d${}^{\prime \prime }$ are used to differentiate between drops)
$d_{32}$	[m]	Sauter mean diameter
$\dot{D}$	$\left[m^{-4}s^{-1}\right]$	death rate in PBE
*D*	[m]	tank diameter
*E*	[J]	energy
*F*	$\left[m^{3}s^{-1}\right]$	coalescence rate
*f*	$\left[m^{-4}\right]$	density function of number
$f_{d}$	[-]	ratio of daughter drop volume to mother drop volume
*g*	$\left[s^{-1}\right]$	breakage rate
*h*	[m]	height
*H*	[m]	height
*n*	$\left[s^{-1}\right]$	stirring speed
$n_{p}$	[-]	number of particles in the system
$n_{d}$	[-]	desorbed number of particles
*P*	$\left[J\;s^{-1}\right]$	power
*q*	$\left[m^{-1}\right]$	density function
*Q*	[-]	cumulative function
*r*	[m]	radius
*s*	[m]	strength
*t*	[s]	time
*T*	[K]	temperature
*V*	$\left[m^{3}\right]$	volume
*w*	[-]	mass fraction
*w*	[m]	wide
**Greek Symbols**
β	[-]	daughter drop size distribution
η	[Pas]	dynamic viscosity
ϵ	$\left[W\;{\mathrm{kg}}^{-1}\right]$	mass specific energy dissipation rate
ε	[-]	voidage
λ	[-]	coalescence efficiency
κ	$\left[S\;m^{-1}\right]$	electrical conductivity
ν	[-]	number of daughter drops
φ	[-]	dispersed-phase volume fraction
ρ	$\left[\mathrm{kg}\;m^{-3}\right]$	density
γ	$\left[N\;m^{-1}\right]$	interfacial tension
$\dot{\gamma }$	$\left[s^{-1}\right]$	shear rate
σ	$\left[m^{3}\right]$	standard deviation
μ	[m]	mean value
θ	${[}^{\circ }]$	contact angle
Θ	[-]	interface coverage degree by particles
ξ	$\left[m^{3}s^{-1}\right]$	coalescence frequency
ζ	[V]	zeta potential
**Subscripts**
ads	adsorption
*b*	breakage
*b*	baffle
*c*	continous phase
*c*	coalescence
cov	covered
*d*	dispersed phase
*d*	desorbed
*d*	daughter
des	desorption
eff	effective
exp	experimental
e/s	between endoscope and stirrer
*g*	gap
in	initial
*m*	mother
max	maximum
min	minimum
np	nanoparticle
*o*	organic phase
o,w	oil/water interface
*p*	particle
sb	stirrer blades
sim	simulated
st	stirrer
stat	in steady-state
tot	total
3	volume-based
