# Supplementary material for: Application of Population Balance Models in Particle-Stabilized Dispersions
Source: Nanomaterials (Basel). 2023 Feb 11;13(4):698. doi: 10.3390/nano13040698 (PMC9964344; doi:10.3390/nano13040698)
Supplement: Supplementary file 1 [file nanomaterials-13-00698-s001.zip › nanomaterials-2197507-supplementary.pdf]

Supplementary material

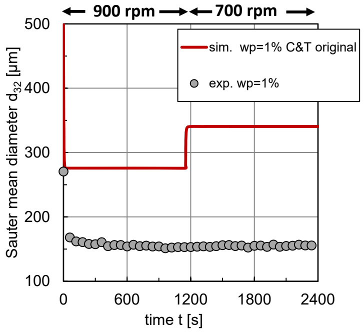

Figure S1. Comparison experimental and simulated transient Sauter mean diameters for $w_p = 1\%$. Simulations were performed with the original (unmodified) C&T submodels and the free parameters summarised in Table 1. The impact of the particles on density and viscosity is considered in the simulations with $\eta_c = 1.72\,mPas$ and $\rho_d = 1003.88\,kg/m^3$.
